# The salivary metabolome of children and parental caregivers in a large-scale family environment study

**DOI:** 10.1038/s44324-024-00024-3

**Published:** 2024-08-13

**Authors:** Jason A. Rothman, Hillary L. Piccerillo, Sage J. B. Dunham, Jenna L. Riis, Douglas A. Granger, Elizabeth A. Thomas, Katrine L. Whiteson

**Affiliations:** 1 Department of Molecular Biology and Biochemistry, University of California, Irvine, Irvine, CA 92697 USA; 2 Institute for Interdisciplinary Salivary Bioscience Research, University of California, Irvine, Irvine, CA USA; 3 Department of Microbiology and Plant Pathology, University of California, Riverside, Riverside, CA USA; 4 Department of Health and Kinesiology, University of Illinois Urbana-Champaign, Urbana, IL USA; 5 Department of Psychological Science, University of California, Irvine, Irvine, CA USA; 6 Department of Pediatrics Johns Hopkins University School of Medicine, Baltimore, MD USA; 7 Department of Neurobiology and Behavior, University of California, Irvine, Irvine, CA USA

**Keywords:** Metabolomics, Metabolomics, Metabolomics

## Abstract

Human metabolism is complex, and is impacted by genetics, cohabitation, diet, health, and environmental inputs. As such, we applied untargeted LC-MS metabolomics to 1425 saliva samples from a diverse group of elementary school-aged children and their caregivers collected during the Family Life Project, of which 1344 were paired into caregiver/child dyads. We compared metabolomes within and between homes, performed population-wide “metabotype” analyses, and measured associations between metabolites and salivary biomeasures of inflammation, antioxidant potential, environmental tobacco smoke (ETS) exposure, metabolic regulation, and heavy metals. Children and caregivers had similar salivary metabolomes, and dyad explained most metabolomic variation. Our data clustered into two groups, indicating that “metabotypes” exist across large populations. Lastly, several metabolites—putative oxidative damage-associated or pathological markers—were correlated with the above-mentioned salivary biomeasures and heavy metals. Implications of the family environment’s effects on metabolomic variation at population, dyadic, and individual levels for human health are discussed.

## Introduction

Metabolomics allows for the assay and quantification of thousands of chemical compounds in a biological sample^1–5^. In humans, metabolomics correlates with the overall metabolism of biochemical pathways in not only the individual, but also microbes that inhabit specific areas of the body^1,6^. Previous studies have shown that metabolomics is a useful tool that has the potential to find biomarkers for diseases and stress, generally through analyzing bodily fluids from subjects^7–9^. One such sample specimen is saliva - a complex matrix of chemical compounds, proteins, and cells important in lubricating the oral cavity, digesting food, and protecting against infection^3,10,11^. As a minimally-invasive biofluid to collect, saliva offers a straightforward way to assay metabolites, proteins, and xenobiotic compounds to find biomarkers for disease or environmental stress exposure^12–14^. Aside from host metabolites, saliva also notably contains microbially-produced compounds and provides a medium to associate host and microbial relationships^1,6,15^. In this study, we concentrate on inter/intrafamily relationships of the salivary metabolome, and associations between this metabolome and biomeasures relating to environmental tobacco smoke (ETS) exposure (the nicotine breakdown product cotinine)^16^, antioxidant potential (uric acid; a compound considered to be a strong antioxidant and is abundant and active in saliva)^12,17,18^, inflammation (C-reactive protein; CRP)^19^, and the modulation of glucose and lipid metabolism (adiponectin; a compound thought to be anti-inflammatory in serum)^13^, along with a selection of metals (chromium, copper, lithium, manganese, and zinc)^14^.

Family members are known to share similar oral health attributes, living environments, and overall diets. Likely due to this close social ecology of the home, several studies have shown that the oral microbiome is more similar within cohabitating family members than those outside of the home^20–24^. Similarly, the overall metabolomes of parental caregivers and their children are more similar than nonfamily individuals, and several metabolites are strongly correlated between family members^25–30^. While the concept of interfamilial metabolic similarity is not novel, many studies concentrate on well-established health biomarkers such as cholesterol, amino acids, and hormones. However, due to the targeted nature of these analytical methods, they likely do not capture overall metabolic relationships within families, and may miss currently-unknown biomarkers of oral health^8,31^. Much like the salivary microbiome^1,20^, the salivary metabolome can be categorized into multiple “ecotypes” of metabolites that may correspond to oral dysbiosis, altered biochemical profiles, or baseline metabolism^1,9,32^, but exactly how widespread this clustering is across large populations remains largely unknown.

The human oral cavity contains a multitude of chemicals derived from host biofluids, and as mentioned above, allows for investigations into the environment in which people live through salivary analyses^10,14^. For example, kit-based spectrophotometric techniques and mass-spectrometry-based methods can detect the presence of analytes involved in nicotine metabolism (i.e., cotinine), drugs and medications, xenobiotics, and metals^14,33,34^. Similarly, through untargeted metabolomics, we may be able to discover novel biomarkers of oral dysbiosis (or, conversely, health) that provide new diagnostic tools and establish baseline measurements of chemicals within saliva^1,3,8,15^. Families living in rural or lower-socioeconomic areas are often exposed to high pollution burdens^14,35,36^, which likely contributes to chronic stress. Furthermore, individuals exposed to heavy metals are known to have higher metabolites associated with oxidative stress and damage (reviewed in Bonvallot 2018^37^. As part of further understanding the effects that environmental stressors have on children^38^, we chose to analyze salivary metals content and associate them with our untargeted metabolomes. Many metals are known to be associated with physiological and neurological problems when observed at high levels^39–41^. These elements serve as reliable indicators of exposure, and we seek to uncover potential biomarkers for metals exposure, which may help us understand unexplored pathways underlying health outcomes, and add to the growing field of “exposomics”^42–44^.

Given the importance of understanding human metabolism and how it relates to families, we investigated the salivary metabolome in the context of children and their caregivers as part of the Family Life Project (FLP), a large-scale prospective longitudinal study^38^. For example, if caregivers are smoking - with negative impacts on oral and other health measures—are these markers shared with children who are not smoking, but share the same household? We asked several questions of our data: First, does the salivary metabolome differ between children and adults (caregivers, henceforth), and is there metabolic concordance within families? Second, does the salivary metabolome associated with biomeasures of ETS exposure, antioxidant potential, metabolic regulation, or inflammation, and are there chemicals in saliva that may serve as additional biomarkers for the above-mentioned biomeasures? Lastly, are there associations between salivary metals burden and metabolites, and might these associations indicate potential negative health outcomes?

## Methods

### Study participants

The Family Life Project (FLP) is a longitudinal study of families residing in Pennsylvania or North Carolina. FLP began in 2003–2004, when a representative sample of 1292 children whose families resided in the target communities at the time the mothers gave birth were recruited and enrolled. Detailed descriptions of the sampling and recruitment procedures are available in Vernon-Feagans, Cox, and the FLP Key Investigators, 2013^38^. Briefly, families with a child born between September 2003 and August 2004 were recruited from hospitals, and multiple subsequent FLP study visits have been conducted with consented participants from children aged 2-months to age 20 years. The current analyses focused on a subset of data collected at the child’s 90-month at-home follow-up visit, where children and their primary caregivers provided unstimulated, resting/baseline saliva samples via passive drool collection. These saliva samples were assayed and archived in −80 ^◦^C freezers, and biospecimens with adequate saliva remaining for metabolome analysis were examined in this study. Out of the total FLP sample, we assessed 1425 saliva samples (Table 1) (child *N* for this subsample = 719; female = 353, male = 366; age = 79–100 months [average = 87 months], caregiver *N* = 706; female = 692, male = 27; age = 22–65 years [average = 34 years) of which 1344 were paired into 672 caregiver/child dyads. Procedures for this study were approved by the Institutional Review Boards of the University of North Carolina (IRB # 07–0646 and 16–2751) and New York University (IRB # IRB-FY2017-69) using deidentified data. For children, informed consent was obtained from the child’s parent or guardian. Informed consent was also obtained from all adult participants in the study. Sample IDs were further randomized prior to analysis and reporting.

**Table 1:** Study sample sizes and relevant demographic information.

Participant	Children	Caregivers
**Total sample number**	719	706
**Sex**	Female = 366 Male = 353	Female = 692 Male = 27
**Average age**	87 months Range = 79–100 months	34 years Range = 22–65 years
**Caregiver self-reported smoking status**	No = 463 Yes = 225 No response = 31	No = 450 Yes = 221 No response = 35
**Biomeasure sample sizes**		
**Adiponectin**	701	NA
**C-reactive protein**	616	NA
**Cotinine**	714	672
**Uric acid**	630	NA
**Chromium**	237	NA
**Copper**	237	NA
**Manganese**	237	NA
**Zinc**	237	NA
**Lithium**	204	NA

### Saliva sample handling for biomeasure analyses

All salivary biomarker analyses were conducted at the UC Irvine Institute for Interdisciplinary Salivary Bioscience Research (IISBR), where samples were stored at −80 °C as previously described^20^. Briefly, we thawed the samples, vortexed, then centrifuged samples for 15 min at 3500 RPM, then analyzed the supernatant for adiponectin, CRP, cotinine, and uric acid concentrations as follows below. We note that only children’s samples were assayed for all biomeasures, while caregiver samples were subject to cotinine analysis only. Samples numbers for each analyte in children were: adiponectin (*N* = 701), CRP (*N* = 616), cotinine (*N* = 714), and uric acid (*N* = 630), while caregivers’ cotinine was measured in (*N* = 672) samples (Table 1).

### Adiponectin, C-reactive protein, cotinine, and uric acid assays

We analyzed salivary adiponectin with the Human Adiponectin MSD assay kit (Meso Scale Discovery, Rockville, MD, USA). We diluted samples fivefold, then assayed following the manufacturer’s supplied protocol using a four-log standard curve and read the sample concentrations on a Meso Quickplex SQ120 spectrophotometer (Meso Scale Discovery, Rockville, MD). Concentrations were derived using MSD Discovery Workbench software v4.0 using curve fit models, with an assay range of sensitivity of 0.06 to 1000 ng/mL.

We assayed CRP in duplicate with the Human CRP V-Plex MSD Multi-spot Assay kit (Meso Scale Discovery, Rockville, MD, USA). We diluted samples five- or ten-fold, then assayed following the manufacturer’s protocol using a modified four-log standard and read the sample concentrations on a Meso Quickplex SQ120 spectrophotometer. The assay range sensitivity was 1.33–46,600 pg/mL CRP.

We measured salivary cotinine concentrations in both children and caregivers using the Salimetrics Salivary Cotinine ELISA kit (Salimetrics, Carlsbad, CA, USA) following the manufacturer’s protocol. We analyzed 20 uL of sample in duplicate by incubating samples with kit reagents for 90 min with shaking at 37 °C, and diluted samples tenfold if subjects reported nicotine use. As above, we washed the plates and then added TMB followed by room temperature incubation for 30 min in the dark. We added 2 M sulfuric acid and read the results on a PowerWave HT spectrophotometer (BioTek/Agilent Technologies, Santa Clara, CA) spectrophotometer, and computed a standard curve using a four-parameter non-linear regression curve fit. The assay range of sensitivity was 0.15 to 200 ng/mL for neat saliva and 1.5 to 2000 ng/mL for tenfold diluted saliva, and we substituted values of ½ the lower limit of measurement for each sample under the lowest reliable measurement for 316 samples (*N* = 138 caregivers and *N* = 178 children).

We analyzed uric acid with the Salimetrics Salivary Uric Acid Assay kit (Salimetrics, Carlsbad, CA, USA). We mixed 10 uL of the sample with 190 uL of uric acid reagent in duplicate following the manufacturer’s protocol, then measured the results on a PowerWave HT spectrophotometer (BioTek/Agilent Technologies, Santa Clara, CA). The uric acid assay had a range of sensitivity from 0.07–20 mg/dL.

### Salivary metals data

We obtained salivary metal concentrations in children from ref. ^14^ and report their methods here for clarity. Briefly, Gatzke-Kopp and colleagues used Inductively Coupled Plasma Optical Emission Spectrometry (ICP-OES) to measure the concentration of metals in aliquots of saliva that we matched to our own. We obtained data from *N* = 237 samples for chromium, copper, manganese, and zinc, while only *N* = 204 samples yielded detectible lithium measurements (Table 1).

### Metabolome sample preparation and mass-spectrometry

We sent frozen saliva samples on dry ice to the West Coast Metabolomics Center (WCMC) at the University of California, Davis, for sample preparation and data collection. Briefly, WCMC extracted 100 uL of saliva with 3:3:2 acetonitrile/isopropanol/water and then evaporated the samples. WCMC ran 5 uL of the sample through hydrophilic interaction chromatography (HILIC) and then used an Agilent 6530 Quadrupole Time of Flight MS/MS mass spectrometer in both positive and negative ion modes to capture ion intensities. WCMC used published protocols to collect data, and ran all samples, quality control pool samples, and method blanks^45–47^ in the same manner and identified mass-spectra using the MassBank of North America database. WCMC normalized the peak areas of samples with Systematic Error Removal Using Random Forest (SERRF)^48^, and we note that within each sample, WCMC added stable isotope “internal control standards” (iSTDs) to the samples before injection to calibrate retention times. We received the data as an Excel file with metabolite names (for identified metabolites only), adducts, ion mode, retention times, mass/charge ratios (m/z), and peak heights for each ion (Dryad dataset^49^). We then assigned “chemical taxonomy” to the named metabolites with the chemical classification software “ClassyFire”^50^.

### Metabolome data analyses

We used R^51^ for data analyses and manipulation. Before any analyses, we removed iSTD ions from the dataset, as the WCMC added these for quality control. We also filtered out ions with an average relative peak height of less than twofold higher in samples or quality control samples as compared to blanks. We normalized the ions to their within-sample relative peak heights and used these values for downstream analyses. Throughout the analyses of the data, we used a “metabology” approach—where metabolomics data are analyzed through community ecology tools to understand the relationships between ions and adequately handle sparse, compositional, multidimensional data^52–54^. We used these data to tabulate Shannon diversity and Bray-Curtis indexes and visualized the data with nonmetric multidimensional scaling ordinations (NMDS) with “ggplot2”^55^ and “patchwork”^56^ with categorical variables self-reported on a questionnaire: “caregiver/child”, “female/male” for children, “smoking/nonsmoking” for caregivers, and “<1 ng/mL or >1 ng/mL” cotinine concentration for children as an estimate for ETS exposure^16^. We tested these variables for statistical significance with Adonis (PERMANOVA with 999 permutations) in the R package “vegan”^57^ for multivariate statistics and linear mixed-effects models on individual named metabolites with “lmerTest”^58^ in R. We obtained salivary metals data from Gatzke-Kopp et al 2023^14^ on sample aliquots from the same original source, and refer to their publication for all relevant materials and methods to metals concentration analyses. Similarly, we assigned metals concentrations to tertile based on concentration within the entire subsample and ran Adonis tests on those data as above.

We used the R package “Hmisc”^59^ to generate Spearman correlations between the relative peak heights of ions and the concentrations of the biomeasures adiponectin, cotinine, CRP, and uric acid, along with each metal for children and cotinine for caregivers only, then corrected the resulting *P* values for multiple comparisons via the Benjamini–Hochberg method. We also used distance-based Redundancy Analysis (db-RDA) in “vegan” to assess the contributions of continuous variables to metabolome variability and plotted the resulting ordinations with “ggplot2.”

Lastly, we performed stomatotype analysis (as in refs. ^20,60,61^) on the metabolomes of paired caregiver/child dyads using Jensen–Shannon distances and partitioning around medoid (PAM) clustering with the R packages “ade4”^62^ and “cluster”^63^. We estimated optimal cluster number by Silhouette values, then used lmerTest for univariate analyses between PAM clusters, adjusted *p* values for multiple comparisons with the Benjamini–Hochberg method, and plotted the resulting PCoA ordination and boxplots of the data with “ggplot2” and “patchwork.”

## Results

We obtained ion profiles from both caregivers (*N* = 706) and children (*N* = 719), along with extraction method blanks (*N* = 153), and quality control pool samples (*N* = 157) (Dryad dataset^49^). From these metabolomes, we detected 2881 unique ions, of which 281 were identified by name, and filtering left us 2046 total ions (both positive and negative ions), of which 210 were named. Overall, the 104 most proportionally represented ions comprised 50.02% of the relative peak heights in our samples (Fig. S1), of which 1409 were present at >0.01% relative peak height. Likewise, identified metabolites only comprised 15.56% of the relative peak heights of ions, while unknown ions accounted for 84.45% (Figs. S1–S4). We used the ClassyFire chemical classifier to assign “chemical taxonomy” to each identified ion and plotted the relative peak heights of the ten most proportionally represented chemical superclasses as stacked bar plots (Figs. S2–S4).

**Fig. 1:**
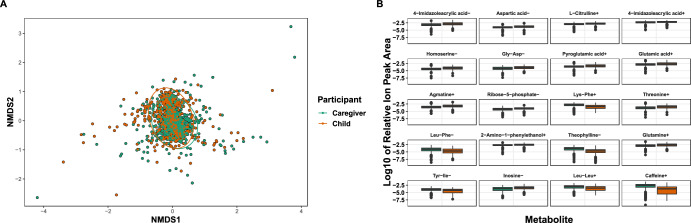
Metabolome diversity differs slightly between children and caregivers. Comparisons of caregivers and children by (**A**) NMDS ordination and (**B**) linear mixed-effects models of the relative peak heights of the 20 most significantly differentially abundant metabolites (*P*_adj_ < 2 × 10^–15^). Dyad explained most of the variation between our subjects (*F* = 1.8, *R*^2^ = 0.62, *P* < 0.001 while categorical age explained ~2% of metabolome variation (*F* = 37.2, *R*^2^ = 0.02, *P* < 0.001). (+) or (−) denotes positive or negative metabolite ions.

We paired 1426 samples into 672 caregiver/child dyads. We compared the diversity of all 2046 ions (as measured by Bray-Curtis dissimilarities) and found that children and caregivers significantly differed (*F* = 37.2, *R*^2^ = 0.02, *P* < 0.001), and dyad explained most of the variation between our subjects (*F* = 1.8, *R*^2^ = 0.62, *P* < 0.001, Fig. 1a), while alpha diversity was not different between caregivers and children (H_(1)_ = 1.6, *P* = 0.20). Then, we used linear mixed-effects models with dyad as a random variable on ions at greater than 0.01% relative peak height and showed that 95 identified metabolites differed between caregivers and children (*P*_adj_ < 0.05, supplemental dataset SF1). Of these, 20 metabolites were highly significantly different between caregivers and children (*P*_adj_ < 2 × 10^−15^): Theophylline-, caffeine+, 4-imidazoleacrylic acid±, ribose-5-phosphate-, inosine-, agmatine+, 2-amino-1-phenylethanol+, and amino acids and their derivatives (Fig. 1 and Supplemental Dataset SF1).

**Fig. 2:**
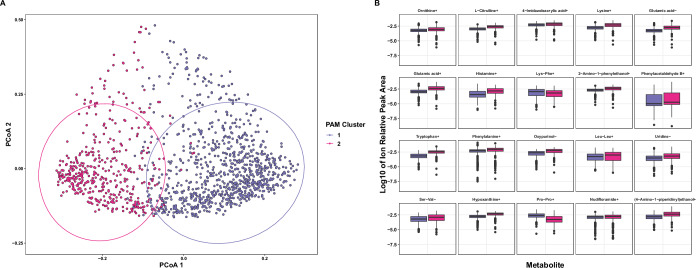
Metabolomes clustered into two overlapping groups (“metabotypes”). Analyses of the metabolomes of paired subjects by (**A**) PAM clustering and (**B**) linear mixed-effects models of the relative peak heights of the 25 most significantly differentially abundant metabolites (*P*_adj_ < 0.001). Only metabolites at greater than 0.1% relative peak heights were analyzed by LMER, and (+) or (−) denotes positive or negative metabolite ions.

We analyzed the metabolomes of paired caregiver/child dyads (*N* = 1344) through PAM clustering and observed that these metabolomes clustered into two overlapping groups of individuals based on Silhouette values (Silhouette = 0.12) and plotted the resulting PCoA ordination (Fig. 2a). Ion beta diversity differed between the two clusters (*F* = 189.6, *R*^2^ = 0.12, *P* < 0.001). We then ran linear mixed-effects models on ions at greater than 0.1% relative peak height with dyad as a random variable to univariately compare the clusters and showed that 25 identified metabolites significantly differed between the clusters (*P*_adj_ < 0.05, Fig. 2b and Supplemental Dataset SF1). Of these, 20 metabolites had *P*_adj_ values of <1 × 10^−6^: Hypoxanthine+, oxypuranol-, 2-amino-1-phenylethanol+, 2-(4-amino-1-piperidinyl)ethanol+, 4-imidazoleacrylic acid+, histamine+, nudifloramide+, phenylacetaldehyde B+, and amino acids and derivatives (including nonproteinogenic) (Fig. 2 and Supplemental Dataset SF1).

**Fig. 3:**
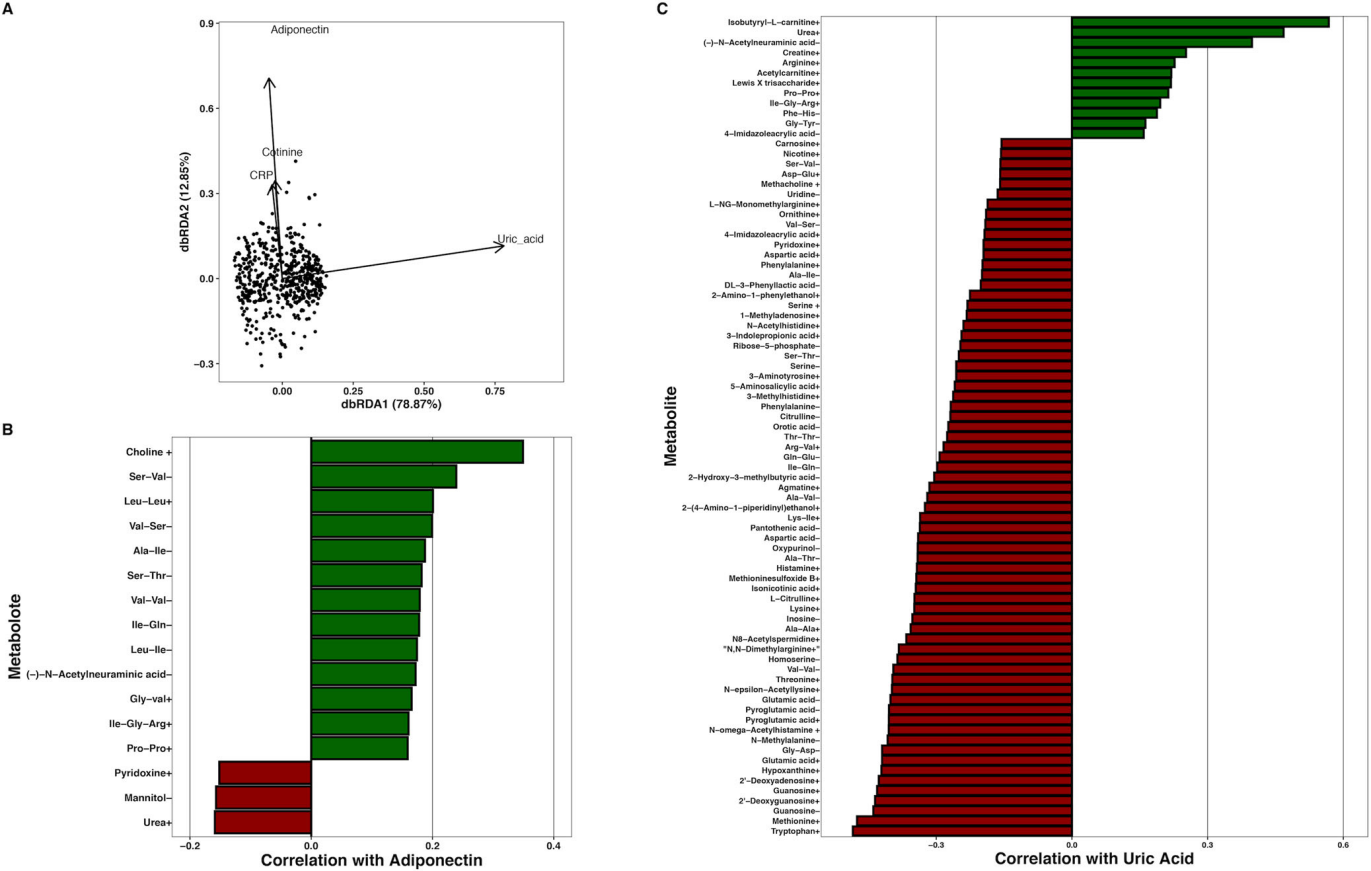
Analyses of children's metabolomes by db-RDA and correlation between metabolites and kit-measured adiponectin and uric acid. **A** db-RDA analysis of children’s metabolomes with kit-assayed biomeasure concentrations overlayed as vectors. Overall, the four measured biomarkers were significantly associated with metabolome separation (*F* = 7.5, *P* < 0.001), with each ion significantly associating with the metabolomes (*P* < 0.026 for each). **B**, **C** Significant Spearman correlations (−0.15 < *ρ* > 0.15 shown only) of identified metabolites with kit-assayed adiponectin and uric acid. (+) or (−) denotes positive or negative metabolite ions.

We were interested in the effects of biological sex on the metabolomes of children, but found no differences between the alpha or beta diversity of boys’ and girls’ overall metabolomes (alpha: [H_(1)_ = 0.44, *P* = 0.51]; beta: F = 1.14, *R*^2^ = 0.002, *P* = 0.25), and did not observe any significant univariate associations between individual ion and sex. We assessed the associations between concentrations of adiponectin, CRP, cotinine, and uric acid with the overall metabolomes of children (*N* = 538) through distance-based redundancy analysis (db-RDA). We observed that the four biomeasures cumulatively explained 5.3% of metabolome variation (Overall *F* = 7.5, *P* < 0.001), with each biomeasure significantly associated with the metabolomes (*P* < 0.026 for each, Fig. 3 and Supplemental Dataset SF1).

We ran univariate Spearman’s correlations on ions at greater than 0.01% average relative peak height to find associations between specific ions and each of the salivary biomeasures examined. We report the sample sizes for each analysis as each biomeasure had a different number of measurements. Concentrations of adiponectin were significantly correlated with 41 metabolites (*N* = 701, −0.16 < ρ < 0.35, *P*_adj_ < 0.05, Fig. 3 and Supplemental Dataset SF1). CRP levels were significantly correlated with 22 metabolites (*N* = 616, −0.13 < ρ < 0.15, *P*_adj_ < 0.05, Fig. 3 and Supplemental Dataset SF1). Lastly, uric acid concentrations were significantly correlated with 94 metabolites (N = 630, −0.49 < ρ < 0.57, *P*_adj_ < 0.05, Fig. 3 and Supplemental Dataset SF1). We also ran these correlation tests on kit-measured cotinine concentrations and reported those results with the smoking/nonsmoking analyses.

**Fig. 4:**
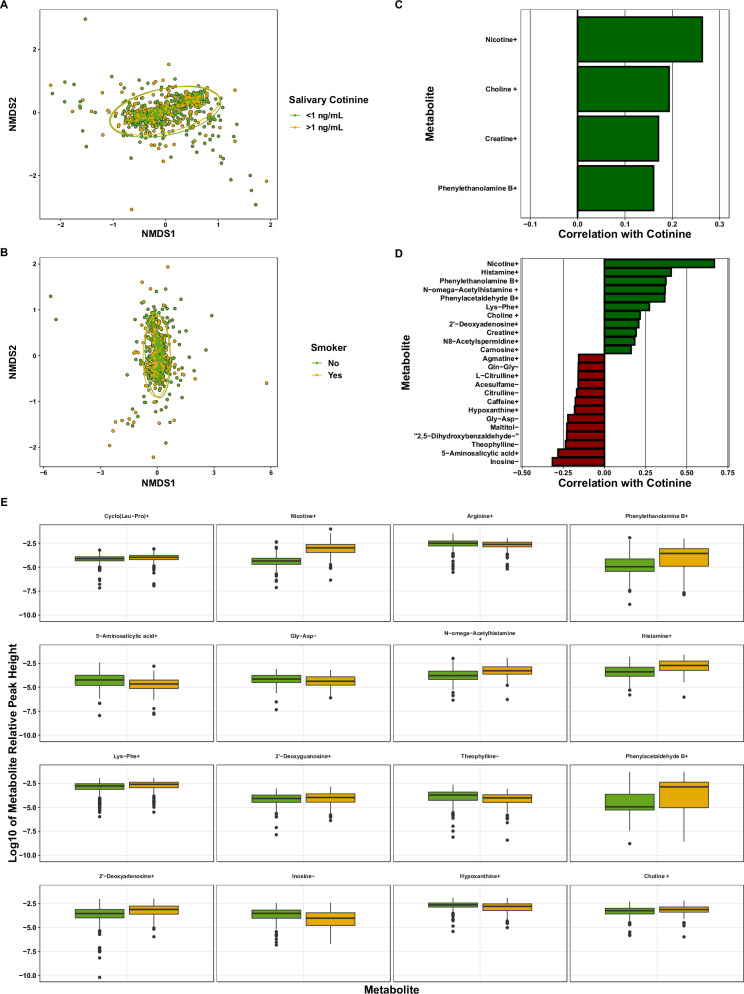
The associations between environmental tobacco smoke exposure and the metabolomes of children and caregivers along with correlations of metabolites and salivary cotinine. **A** NMDS ordination of the metabolomes of children with salivary cotinine <1 ng/uL versus >1 ng/uL and **B** caregivers who reported smoking or not. ETS exposure did not significantly alter the metabolome of children (*F* = 1.1, *R*^2^ = 0.002, *P* = 0.29), but did affect caregivers’ (*F* = 8.6, *R*^2^ = 0.01, *P* < 0.001. **C**, **D** Significant Spearman correlations (*P*_adj_ < 0.05, (−0.15 < *ρ* > 0.15 shown only) of salivary cotinine and kit-measured cotinine in (**C**) children or **D** caregivers. **E** Boxplots of the relative peak heights of significantly different metabolites between smoking and nonsmoking caregivers as tested by generalized linear models (*P*_adj_ < 0.05, only *P*_adj_ < 0.01 shown). (+) or (−) denotes positive or negative metabolite ions.

We compared the associations between ETS exposure and children’s (*N* = 714) and caregivers’ (*N* = 672) metabolomes separately because of the different routes of smoking exposure. We analyzed children’s metabolomes by categorically classifying children by their kit-measured cotinine concentration (children’s cotinine levels <1 ng/uL and >1 ng/uL) but did not see a significant relationship between ETS exposure and metabolic alpha (H_(1)_ = 0.25, *P* = 0.61) or beta diversity (*F* = 1.1, *R*^2^ = 0.002, *P* = 0.29, Fig. 4a). We then ran generalized linear models on each metabolite and found that only nicotine+ was significantly different between groups (*P*_adj_ < 0.001, Supplemental Dataset SF1).

As the intensity of ETS exposure likely causes different effects on the metabolome we ran Spearman correlations between kit-assayed cotinine and individual metabolites in children (*N* = 714). Cotinine was significantly correlated with 14 metabolites (−0.11 < ρ < 0.26, *P*_adj_ < 0.05, Fig. 4 and Supplemental Dataset SF1).

We found that self-reported smoking status slightly related to the metabolome beta diversity of caregivers (*F* = 8.6, *R*^2^ = 0.01, *P* < 0.001, Fig. 4), but not alpha diversity (H_(1)_ = 0.44, *P* = 0.51). We used generalized linear models on individual ions at greater than 0.01% relative peak height to find associations with caregivers’ smoking status. Only 22 identified metabolites significantly differed between groups: Nicotine+, 4-imidazoleacrylic acid+, phenylethanolamine B+, 5-aminosalicylic acid+, *N*-omega-acetylhistamine+, histamine+, 2’-deoxyguanosine+, theophylline-, phenylacetaldehyde B+, 2’-deoxyadenosine+, inosine-, hypoxanthine+, choline+, nudifloramide+, and amino acids and derivatives (Fig. 4 and Supplemental Dataset SF1).

We ran Spearman correlations between individual ions and kit-measured cotinine in caregivers in the same fashion as above in children. Cotinine was significantly correlated with 39 identified metabolites (−0.32 < ρ < 0.67, *P*_adj_ < 0.05, Fig. 4 and Supplemental Dataset SF1).

**Fig. 5:**
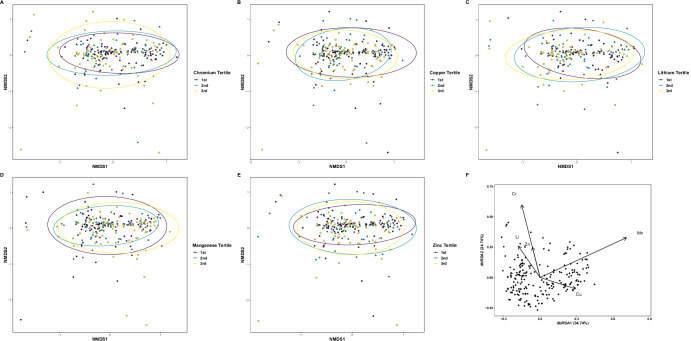
Metabolomic diversity and db-RDA analysis of children's metabolomes and salivary metals. NMDS ordinations of metabolomic diversity as a function of salivary metal tertiles for (**A**) chromium, **B** copper, **C** lithium, **D** manganese, and **E** zinc for each metal tested. Metals significantly associated with metabolome beta diversity ([Li: *R*^2^ = 0.01, *F* = 1.5, *P* = 0.044], Cr: [*R*^2^ = 0.01, *F* = 1.5, *P* = 0.039], Cu: [*R*^2^ = 0.02, *F* = 2.1, *P* = 0.004], Mn: [*R*^2^ = 0.02, *F* = 2.7, *P* < 0.001], Zn: [*R*^2^ = 0.01, *F* = 1.6, *P* = 0.038]). Panel (**F**) is a db-RDA of all metal concentrations. Metals explained 4.6% of metabolome variation (Overall *F* = 1.9, *P* < 0.001), and chromium, manganese, and copper contributed significantly to the variation, while lithium and zinc did not (Cr, Mn, Cu: *P* < 0.05, Li and Zn: *P* > 0.05).

We measured the associations between the metals lithium (*N* = 204; average = 13.9 μg/L, range = 0.11–845.5 μg/L), chromium (*N* = 237; average = 7.3 μg/L, range = 0.07–22.5 μg/L), copper (*N* = 237; average = 29.0 μg/L, range = 0.23–844.2 μg/L), manganese (*N* = 237; average = 8.6 μg/L, range = 0.04–122.5 μg/L), and zinc (*N* = 237; average = 60.9 μg/L, range = 0.09–566.0 μg/L) measured in saliva and the metabolomes in children. As the metals were reported as concentrations (ug/L), we split the metals results into tertiles (i.e., 1^st^, 2^nd^, 3^rd^) to analyze the associations categorically. Salivary metal tertile for each element significantly associated with metabolome beta diversity ([Li: *R*^2^ = 0.01, *F* = 1.5, *P* = 0.044], Cr: [*R*^2^ = 0.01, *F* = 1.5, *P* = 0.039], Cu: [*R*^2^ = 0.02, *F* = 2.1, *P* = 0.004], Mn: [*R*^2^ = 0.02, *F* = 2.7, *P* < 0.001], Zn: [*R*^2^ = 0.01, *F* = 1.6, *P* = 0.038], Fig. 5), but not metabolome alpha diversity (*P* > 0.05 for each, Fig. 5). We then used db-RDA on the samples with measured concentrations of all metals (*N* = 204) and found that these metals explained 4.6% of metabolome variation (Overall *F* = 1.9, *P* < 0.001), but only chromium, manganese, and copper contributed meaningfully to this variation, while lithium and zinc did not (Cr, Mn, Cu: *P* < 0.05, Li and Zn: *P* > 0.05, Fig. 5 and Supplemental Dataset SF1).

**Fig. 6:**
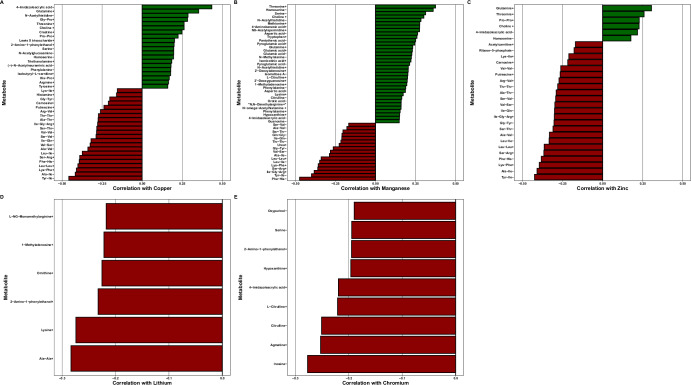
Metabolite/metals correlation analyses. Significant Spearman correlations between metals concentration and individual metabolites at greater than 0.01% relative peak height (*P*_adj_ < 0.05) for (**A**) copper, **B** manganese, **C** zinc, **D** lithium, and **E** chromium. (+) or (−) denotes positive or negative metabolite ions.

We also measured univariate Spearman correlations between metals concentration and individual metabolites at greater than 0.01% relative peak height. Lithium was significantly correlated with six metabolites (ρ < −0.21, *P*_adj_ < 0.05, Fig. 6, Supplemental Dataset SF1), chromium was significantly correlated with nine metabolites (ρ < −0.18, *P*_adj_ < 0.05, Fig. 6, Supplemental Dataset SF1), copper was significantly correlated with 42 metabolites (−0.46 < ρ < 0.44, *P*_adj_ < 0.05, Fig. 6, Supplemental Dataset SF1), manganese was significantly correlated with 52 metabolites (−0.47 < ρ < 0.38, *P*_adj_ < 0.05, Fig. 6, Supplemental Dataset SF1), and zinc significantly correlated with 29 metabolites (−0.43 < ρ < 0.31, *P*_adj_ < 0.05, Fig. 6, Supplemental Dataset SF1).

## Discussion

Through our exploratory analyses of a large-scale sample from the 90-month assessment of the Family Life Project cohort^38^, we were able to assay the salivary metabolome and its associations with biomeasures relating to antioxidant potential, environmental tobacco smoke (ETS) exposure, and systemic inflammation. Most of the variance in salivary metabolomes were explained by family dyad, supporting the view that home environments are incredibly impactful on people, and the closeness of families often result in similar metabolisms and health measures. We also find that a small but significant proportion of the variance is explained by how children’s and adults’ metabolomes differed overall^25–29^. Extending this concept further, subjects generally clustered into two overlapping metabolomic groups, suggesting that people fall into sub-groups with shared features, although we are unable to discern exactly what causes this grouping^1^. We observed significant but minor shifts in smokers’ metabolomes, but not in their children, indicating that primary ETS exposure can affect salivary metabolites and may be associated with increased inflammation and polyamine turnover^4,33,64,65^. We also show that kit-based biomeasures correspond well with metabolites intermediate in their respective biochemical pathways, suggesting both kit-based spectrophotometry and mass-spectrometry-based approaches are useful to assaying metabolism. Lastly, salivary metals were associated with altered children’s metabolomes, and were often anticorrelated with putative proteolysis products, suggesting that environmental metals may affect metabolism^66^, and that saliva is a useful biofluid in which to assay metals^14^. Collectively, our study represents a large-scale investigation into the metabolism of families and how these chemicals may relate to cohabitating individuals.

Our study shows that the caregiver/child dyad explained most of the salivary metabolome variation, supporting others’ similar findings in specific metabolites^25–28^, but previously unknown in large-scale studies measuring the overall metabolome. Many factors are known to affect metabolism, such as age, sex, diet, and, increasingly, familial environment, and our work adds an important dimension to this metabolic variability^1,28,67^. By applying untargeted metabolomics, we can start to unravel the complex interactions that family and social structure has on the metabolism of individuals. Even though the child/caregiver dyad explained the vast majority of metabolic variation, several ions’ relative peak heights differed between children and caregivers - many of which are “lifestyle,” medicinal, or environmental molecules. Chemicals such as nicotine, caffeine, theophylline, phenylacetaldehyde, acetaminophen, and salicylic acid were all higher in adults, likely due to diet or medication^4^. Similarly, adults generally had higher relative peak areas of dipeptides (suggesting proteolysis or oxidative damage), while children had more ions basal or intermediate to chemical pathways, perhaps due to children’s higher basal metabolic rate and growth. As amino acid metabolism has been shown to change with age^4^ and differs across populations^1^, more research is needed to understand amino acid metabolism associations with medical conditions or normal development. We also note that large proportions of our data consist of unidentified ions and are likely important to a discerning child from a caregiver, but we are unable to classify these peaks and cannot rule out that the observed spectral peaks are not distinct ions. We appreciate that the magnitude of these differences, while significant, are slight, further stressing the impact and importance of family (relatedness and/or cohabitation) on metabolism, and that our sample population was targeted to specific locations and demographics, reducing the generalizability of our results. We also recognize that our metabolomics data are biased by only using one type of chromatography, which likely overrepresents biogenic amines at the cost of other ion species^68^, and that matrix effects and ionization efficiencies may impact the interpretation of our results^69^.

Unsupervised clustering of large-scale, multidimensional metabolomics data has been used to group diseased and healthy individuals by common metabolic patterns and provide insights into their underlying physiology^1,9,32,70–72^. In our study, subjects’ metabolomes tended to cluster into two overlapping groups which did not obviously correspond to any of our metadata categories (i.e., sex, age, smoking, state of residence, etc.). These clusters were somewhat separated by free dipeptides, a Lewis X trisaccharide (Le^X^) and intermediates of the urea cycle (i.e., citrulline, arginine, and ornithine), but mainly differed by the proportion of unidentified, ions and we note that the overall differences between clusters were relatively minor as seen before^1^. Previous cluster-based metabolomics studies have shown that differences in amino acid metabolism can cause cluster separation^1,9,32^, and oral microorganisms impact the concentrations of these metabolites^1,15^, so future studies should incorporate “multi-‘omic” approaches to understanding the chemical and microbial ecology of the mouth. Likewise, urea cycle metabolites were important in cluster separation. Intermediates of the urea cycle have been implicated as potential indicators for diverse maladies, such as hypertension^73^, breast cancer^32^, severe inflammation^74^, and urea cycle disorders^75^, suggesting that untargeted metabolomics and cluster analyses are useful to find compounds that may serve as biomarkers for disease. While the clusters largely separate in ordination space, we note that there is substantial overlap, indicating variability and noise in our data, which may be affecting our interpretations. Similarly, as we are unable to pinpoint exactly what is driving metabolome clustering, our results may be influenced by diet or other personal choices that are not reflected in our metadata^1,2,4,15,76,77^, or unknown interactions/ion detection phenomena^69^.

Adiponectin, CRP, and uric acid are significantly associated with altered metabolomes in children, with uric acid explaining the most variance. As the endpoint in purine degradation^78^, uric acid was (as expected) strongly anticorrelated with intermediates of purine metabolism. Conversely, acylcarnitines were correlated with uric acid, which may indicate consumption of a high-fat/high-protein diet and subsequent lipid accumulation^79^, and an increase in creatine with uric acid may also be due to protein intake and subsequent purine metabolism or antioxidant activity^80,81^. We found many positive correlations between free dipeptides and adiponectin—a molecule predicted to modulate inflammation and oxidative stress^13,82^. As dipeptides are thought to be biomarkers for proteolysis^83^ and may have antioxidant activity^84^ the correlations of adiponectin and dipeptides could indicate infections or inflammation in children’s mouths, and so may serve as a useful salivary biomeasure for immune activity. Lastly, CRP had very slight associations with overall salivary metabolomes and individual metabolites, suggesting that while CRP is a useful biomarker for systemic inflammation, it is also nonspecific^85^, and may not associate well with metabolic pathways in saliva^15^. Collectively, these results suggest that the spectrophotometry kit-based biomeasures capture useful physiological markers for their respective pathways or functions, and indicate that saliva is a worthwhile biospecimen for assaying these measures^10^.

Environmental tobacco smoke (ETS) exposure is a major, worldwide cause of morbidity and has been shown to alter human metabolism^4,33,64^. Smoking minorly affected caregivers’ overall metabolomes, but exposure to ETS did not alter children’s—likely because children were only exposed passively and did not receive the same nicotine dose as active smokers, although this is speculative because we do not have survey data reflecting total nicotine consumption. In both children and caregivers, several metabolites were correlated with kit-assayed cotinine (the major final metabolite of nicotine), including phenylethanolamine, phenylacetaldehyde, choline, creatine, *N*8-acetylspermidine, and unsurprisingly nicotine, along with a strong apparent histamine response in caregivers. As an agonist of acetylcholine receptors, nicotine has been shown to increase acetylcholine demand (and therefore choline catabolized from phospholipids)^86^, which may be driving our perceived proportional increase in salivary choline^87^, along with increasing phenylethanolamine *N*-methyltransferase activity (possibly observed in our study as increased phenylethanolamine)^88^. Likewise, smoking reduces creatine kinase activity (converts creatine to phosphocreatine), which may cause higher levels of creatine with increasing nicotine concentrations^89,90^. In regard to *N*8-acetylspermine—a molecule indicative of polyamine turnover and associated with vascular pathologies^65,91^—again, we observed a correlation with nicotine. Lastly, histamine was markedly correlated to cotinine concentration, suggesting that histamine production, mast cell activations, and inflammation are affected by nicotine use in caregivers^92,93^. While the overall metabolomic associations with self-reported smoking status and cotinine concentration were minor, the above individual correlations indicate that saliva metabolomics are useful to find potential biomarkers that may indicate altered physiology related to tobacco smoke exposure^4,33,64^.

Salivary metal concentrations were slightly associated with altered child metabolomes, suggesting that metal consumption/exposure potentially affects metabolism^34,94–97^. As the metals cooccurred in the samples, we analyzed them simultaneously, and show that there are likely synergistic associations with the metabolome, which shifts the individuals’ overall metabolism^96^. When specifically considering zinc, copper, and manganese, metals concentration were often anticorrelated with free dipeptides—potential markers for proteolysis and oxidative stress—suggesting that these metals may act as antioxidants reducing the body’s need to produce its own dipeptide antioxidants^83,84^. Conversely, there were positive associations between these metals and free amino acids, which may indicate a shift toward complete protein degradation or proteosome activation^66,97^. Taken together, these results suggest that metals are involved in protein metabolism or that metals may be acting as or along with antioxidants, but more research into oral metabolomics is needed, and we recognize that our results are correlative. Still, our work suggests that metals exposure and the metabolome interact, and more studies should incorporate these types of analyses to better understand human metabolism.

## Conclusions

Metabolomics allows for the simultaneous detection of thousands of chemicals and can provide insights into the complex biochemical activity of human metabolism. Our study suggests that there are both large-scale patterns and subtle differences in the salivary metabolome between populations and within families, and that cohabitation likely affects metabolism. Taken together, metabolomics and kit-based biomeasure analyses indicate that tobacco smoke affects primary users’ metabolism, and that there are several putative biomeasures for antioxidant potential, tobacco smoke ETS exposure, and systemic inflammation along with metals concentrations, that can be studied for further use in understanding the biochemistry of environmental exposures and stress. As our study is mainly discovery-based, we suggest that future research investigate the interactions between the oral microbiome and metabolome in a more targeted way, and that “multi-omic” approaches be applied to family-based or large-populational studies to understand the complex microbes and molecules that underly human health.

## Data Availability

Tabulated mass-spectrometry metabolomics data are freely available on Dryad (10.5061/dryad.66t1g1k88)^49^.

## Supplemental_information

supplemental_dataset
Supplemental_information

## References

[1] Zaura, E. et al. On the ecosystemic network of saliva in healthy young adults. *ISME J* **11**, 1218–1231 (2017). 28072421 10.1038/ismej.2016.199 PMC5475835

[2] Bar, N. et al. A reference map of potential determinants for the human serum metabolome. *Nature*10.1038/s41586-020-2896-2 (2020). 33177712 10.1038/s41586-020-2896-2

[3] Dame, Z. T. et al. The human saliva metabolome. *Metabolomics* **11**, 1864–1883 (2015).

[4] Dunn, W. B. et al. Molecular phenotyping of a UK population: defining the human serum metabolome. *Metabolomics* **11**, 9–26 (2015). 25598764 10.1007/s11306-014-0707-1 PMC4289517

[5] De Filippis, F. et al. The same microbiota and a potentially discriminant metabolome in the saliva of omnivore, ovo-lacto-vegetarian and vegan individuals. *PLoS ONE* **9**, e112373 (2014). 25372853 10.1371/journal.pone.0112373 PMC4221475

[6] Nguyen, T. et al. Host-microbe interactions: profiles in the transcriptome, the proteome, and the metabolome. *Periodontol. 2000* **82**, 115–128 (2020). 31850641 10.1111/prd.12316 PMC7968888

[7] Bertram, H. C., Eggers, N. & Eller, N. Potential of human saliva for nuclear magnetic resonance-based metabolomics and for health-related biomarker identification. *Anal. Chem.* **81**, 9188–9193 (2009). 19780580 10.1021/ac9020598

[8] Gardner, A., Carpenter, G. & So, P.-W. Salivary metabolomics: from diagnostic biomarker discovery to investigating biological function. *Metabolites* **10**, 2 (2020). 10.3390/metabo10020047 PMC7073850 31991929

[9] Su, M.-W. et al. Blood multiomics reveal insights into population clusters with low prevalence of diabetes, dyslipidemia and hypertension. *PLoS ONE* **15**, e0229922 (2020). 32134946 10.1371/journal.pone.0229922 PMC7058291

[10] Granger, D. A. & Taylor, M. K. *Salivary Bioscience: Foundations of Interdisciplinary Saliva Research and Applications* (Springer International Publishing, 2020).

[11] Maughan, H. & Whiteson, K. in *Salivary Bioscience: Foundations of Interdisciplinary Saliva Research and Applications* (eds. Granger, D. A. & Taylor, M. K.) Ch. 7 (Springer International Publishing, 2020).

[12] Riis, J. L. et al. The validity, stability, and utility of measuring uric acid in saliva. *Biomark. Med.* **12**, 583–596 (2018). 29873515 10.2217/bmm-2017-0336 PMC6479278

[13] Riis, J. L. et al. Adiponectin: Serum-saliva associations and relations with oral and systemic markers of inflammation. *Peptides* **91**, 58–64 (2017). 28363793 10.1016/j.peptides.2017.03.006

[14] Gatzke-Kopp, L. M. et al. Environmental tobacco smoke exposure is associated with increased levels of metals in children’s saliva. *J. Expo. Sci. Environ. Epidemiol*. 10.1038/s41370-023-00554-w. (2023) 10.1038/s41370-023-00554-w PMC10733142 37147431

[15] Tang, Z.-Z. et al. Multi-omic analysis of the microbiome and metabolome in healthy subjects reveals microbiome-dependent relationships between diet and metabolites. *Front. Genet.* **10**, 454 (2019). 31164901 10.3389/fgene.2019.00454 PMC6534069

[16] Benowitz, N. L. Biomarkers of environmental tobacco smoke exposure. *Environ. Health Perspect.* **107**, 349–355 (1999). 10350520 10.1289/ehp.99107s2349 PMC1566286

[17] Becker, B. F. Towards the physiological function of uric acid. *Free Radic. Biol. Med.* **14**, 615–631 (1993). 8325534 10.1016/0891-5849(93)90143-i

[18] Battino, M., Ferreiro, M. S., Gallardo, I., Newman, H. N. & Bullon, P. The antioxidant capacity of saliva: The antioxidant capacity of saliva. *J. Clin. Periodontol.* **29**, 189–194 (2002). 11940135 10.1034/j.1600-051x.2002.290301x.x

[19] Paraskevas, S., Huizinga, J. D. & Loos, B. G. A systematic review and meta-analyses on C-reactive protein in relation to periodontitis. *J. Clin. Periodontol.* **35**, 277–290 (2008). 18294231 10.1111/j.1600-051X.2007.01173.x

[20] Rothman, J. A. et al. Oral microbial communities in children, caregivers, and associations with salivary biomeasures and environmental tobacco smoke exposure. *mSystems* **8**, e0003623 (2023). 37338237 10.1128/msystems.00036-23 PMC10470043

[21] Lira-Junior, R., Åkerman, S., Klinge, B., Boström, E. A. & Gustafsson, A. Salivary microbial profiles in relation to age, periodontal, and systemic diseases. *PLoS ONE* **13**, e0189374 (2018). 29538390 10.1371/journal.pone.0189374 PMC5851536

[22] Cephas, K. D. et al. Comparative analysis of salivary bacterial microbiome diversity in edentulous infants and their mothers or primary care givers using pyrosequencing. *PLoS ONE* **6**, e23503 (2011). 21853142 10.1371/journal.pone.0023503 PMC3154475

[23] Burcham, Z. M. et al. Patterns of oral microbiota diversity in adults and children: a crowdsourced population study. *Sci. Rep.* **10**, 2133 (2020). 32034250 10.1038/s41598-020-59016-0 PMC7005749

[24] Foxman, B. et al. The effects of family, dentition, and dental caries on the salivary microbiome. *Ann. Epidemiol.* **26**, 348–354 (2016). 27157862 10.1016/j.annepidem.2016.03.006 PMC5015694

[25] Andraos, S. et al. Population epidemiology and concordance for plasma amino acids and precursors in 11-12-year-old children and their parents. *Sci. Rep.* **11**, 3619 (2021). 33574360 10.1038/s41598-020-80923-9 PMC7878730

[26] Andraos, S. et al. Plasma trimethylamine N-oxide (TMAO) and its precursors: population epidemiology, parent-child concordance, and associations with reported dietary intake in 11-12-year-old children and their parents. *Curr. Dev. Nutr.*10.1093/cdn/nzaa103. (2020) 10.1093/cdn/nzaa103 PMC7335361 32666035

[27] Andraos, S. et al. Plasma B vitamers: population epidemiology and parent-child concordance in children and adults. *Nutrients* **13**, 821 (2021). 33801409 10.3390/nu13030821 PMC8001009

[28] Voerman, E. et al. A population-based resource for intergenerational metabolomics analyses in pregnant women and their children: the Generation R Study. *Metabolomics* **16**, 43 (2020). 32206914 10.1007/s11306-020-01667-1 PMC7089886

[29] Ellul, S. et al. Metabolomics: population epidemiology and concordance in Australian children aged 11-12 years and their parents. *BMJ Open* **9**, 106–117 (2019). 31273021 10.1136/bmjopen-2017-020900 PMC6624050

[30] Foxman, B. et al. Exploring the effect of dentition, dental decay and familiality on oral health using metabolomics. *Infect. Genet. Evol.* **22**, 201–207 (2014). 24080168 10.1016/j.meegid.2013.09.020 PMC3943654

[31] Peisl, B. Y. L., Schymanski, E. L. & Wilmes, P. Dark matter in host-microbiome metabolomics: Tackling the unknowns-A review. *Anal. Chim. Acta* **1037**, 13–27 (2018). 30292286 10.1016/j.aca.2017.12.034

[32] Gal, J. et al. Comparison of unsupervised machine-learning methods to identify metabolomic signatures in patients with localized breast cancer. *Comput. Struct. Biotechnol. J.* **18**, 1509–1524 (2020). 32637048 10.1016/j.csbj.2020.05.021 PMC7327012

[33] Gu, F. et al. Cigarette smoking behaviour and blood metabolomics. *Int. J. Epidemiol.* **45**, 1421–1432 (2016). 26721601 10.1093/ije/dyv330 PMC5100605

[34] Papaioannou, N. et al. Multi-omics analysis reveals that co-exposure to phthalates and metals disturbs urea cycle and choline metabolism. *Environ. Res.* **192**, 110041 (2021). 32949613 10.1016/j.envres.2020.110041

[35] Jones, D. H., Yu, X., Guo, Q., Duan, X. & Jia, C. Racial disparities in the heavy metal contamination of urban soil in the southeastern United States. *Int. J. Environ. Res. Public Health* **19**, 1105 (2022). 35162130 10.3390/ijerph19031105 PMC8834334

[36] Masri, S. et al. Risk assessment of soil heavy metal contamination at the census tract level in the city of Santa Ana, CA: implications for health and environmental justice. *Environ. Sci. Process. Impacts* **23**, 812–830 (2021). 33954329 10.1039/d1em00007a PMC8224146

[37] Bonvallot, N. et al. Metabolomics as a powerful tool to decipher the biological effects of environmental contaminants in humans. *Curr. Opin. Toxicol.* **8**, 48–56 (2018).

[38] Vernon-Feagans, L., Cox, M. & Key, F. L. F. The family life project: an epidemiological and developmental study of young children living in poor rural communities. *Monogr. Soc. Res. Child Dev.* **78**, 1–150 (2013). 24147448 10.1111/mono.12046

[39] Guilarte, T. R. Manganese neurotoxicity: new perspectives from behavioral, neuroimaging, and neuropathological studies in humans and non-human primates. *Front. Aging Neurosci.* **5**, 23 (2013). 23805100 10.3389/fnagi.2013.00023 PMC3690350

[40] Rechtman, E. et al. Sex-specific associations between co-exposure to multiple metals and visuospatial learning in early adolescence. *Transl. Psychiatry* **10**, 358 (2020). 33087698 10.1038/s41398-020-01041-8 PMC7578810

[41] Chasapis, C. T., Ntoupa, P.-S. A., Spiliopoulou, C. A. & Stefanidou, M. E. Recent aspects of the effects of zinc on human health. *Arch. Toxicol.* **94**, 1443–1460 (2020). 32394086 10.1007/s00204-020-02702-9

[42] Wei, S. et al. Metabolomics as a valid analytical technique in environmental exposure research: application and progress. *Metabolomics* **18**, 35 (2022). 35639180 10.1007/s11306-022-01895-7

[43] Sun, J. et al. A review of environmental metabolism disrupting chemicals and effect biomarkers associating disease risks: where exposomics meets metabolomics. *Environ. Int.* **158**, 106941 (2022). 34689039 10.1016/j.envint.2021.106941

[44] Bessonneau, V., Pawliszyn, J. & Rappaport, S. M. The saliva exposome for monitoring of individuals’ health trajectories. *Environ. Health Perspect.* **125**, 077014 (2017). 28743678 10.1289/EHP1011 PMC5801473

[45] Cajka, T. & Fiehn, O. Toward merging untargeted and targeted methods in mass spectrometry-based metabolomics and lipidomics. *Anal. Chem.*10.1021/acs.analchem.5b04491 (2016). 10.1021/acs.analchem.5b04491 26637011

[46] Kind, T. et al. Identification of small molecules using accurate mass MS/MS search. *Mass Spectrom. Rev.*10.1002/mas.21535 (2018). 10.1002/mas.21535 PMC8106966 28436590

[47] Kind, T. et al. FiehnLib: mass spectral and retention index libraries for metabolomics based on quadrupole and time-of-flight gas chromatography/mass spectrometry. *Anal. Chem*. 10.1021/ac9019522 (2009). 10.1021/ac9019522 PMC2805091 19928838

[48] Fan, S. et al. Systematic error removal using random forest for normalizing large-scale untargeted lipidomics data. *Anal. Chem.* **91**, 3590–3596 (2019). 30758187 10.1021/acs.analchem.8b05592 PMC9652764

[49] Rothman, J. et al. Data for: The salivary metabolome of children and parental caregivers in a large-scale family environment study. Dryad dataset 10.5061/dryad.66t1g1k88 (2024). 10.1038/s44324-024-00024-3 PMC12118755 40603689

[50] Djoumbou Feunang, Y. et al. ClassyFire: automated chemical classification with a comprehensive, computable taxonomy. *J. Cheminform.* **8**, 61 (2016). 27867422 10.1186/s13321-016-0174-y PMC5096306

[51] R Core Team. R: a language and environment for statistical computing. (R Foundation for Statistical Computing, 2016).

[52] Dunham, S. J. B. et al. Sex-specific associations between AD genotype and the microbiome of human amyloid beta knock-in (hAβ-KI) mice. *Alzheimers Dement*. 10.1002/alz.13794 (2024). 10.1002/alz.13794 PMC11247698 38572865

[53] Passos Mansoldo, F. R., Garrett, R., da Silva Cardoso, V., Alves, M. A. & Vermelho, A. B. Metabology: analysis of metabolomics data using community ecology tools. *Anal. Chim. Acta* **1232**, 340469 (2022). 36257759 10.1016/j.aca.2022.340469

[54] Dunham, S. J. B. et al. Longitudinal analysis of the microbiome and metabolome in the 5xfAD mouse model of Alzheimer’s Disease. *MBio* **13**, e0179422 (2022). 36468884 10.1128/mbio.01794-22 PMC9765021

[55] Wickham, H. *Ggplot2: Elegant Graphics for Data Analysis* (Springer-Verlag, 2009).

[56] Pedersen, T. L. Patchwork: the composer of plots. *R package version* **1**, 182 (2020).

[57] Oksanen, J. et al. Vegan: community ecology package. (2017).

[58] Kuznetsova, A., Brockhoff, P. B. & Christensen, R. H. B. lmerTest package: tests in linear mixed effects models. *J. Stat. Softw.* **82**, 1–26 (2017).

[59] Harrell, F. E. Hmisc: Harrell Miscellaneous*.* (2019).

[60] Willis, J. R. et al. Citizen science charts two major “stomatotypes” in the oral microbiome of adolescents and reveals links with habits and drinking water composition. *Microbiome* **6**, 218 (2018). 30522523 10.1186/s40168-018-0592-3 PMC6284318

[61] Arumugam, M. et al. Enterotypes of the human gut microbiome. *Nature* **473**, 174–180 (2011). 21508958 10.1038/nature09944 PMC3728647

[62] Dray, S. & Dufour, A. B. The ade4 package: implementing the duality diagram for ecologists. *J. Stat. Softw* **22**, 1–20 (2007).

[63] Maechler, M., Rousseeuw, P., Struyf, A., Hubert, M. & Hornik, K. Cluster: cluster analysis basics and extensions. (2019).

[64] Hsu, P.-C. et al. Metabolomic profiles of current cigarette smokers. *Mol. Carcinog.* **56**, 594–606 (2017). 27341184 10.1002/mc.22519 PMC5646689

[65] Nayak, A. et al. N8-Acetylspermidine: a polyamine biomarker in ischemic cardiomyopathy with reduced ejection fraction. *J. Am. Heart Assoc.* **9**, e016055 (2020). 32458724 10.1161/JAHA.120.016055 PMC7429012

[66] Tamás, M. J., Sharma, S. K., Ibstedt, S., Jacobson, T. & Christen, P. Heavy metals and metalloids as a cause for protein misfolding and aggregation. *Biomolecules* **4**, 252–267 (2014). 24970215 10.3390/biom4010252 PMC4030994

[67] Saben, J. L., Sims, C. R., Piccolo, B. D. & Andres, A. Maternal adiposity alters the human milk metabolome: associations between nonglucose monosaccharides and infant adiposity. *Am. J. Clin. Nutr.* **112**, 1228–1239 (2020). 32844207 10.1093/ajcn/nqaa216

[68] Konieczna, L. et al. Analytical approach to determining human biogenic amines and their metabolites using eVol microextraction in packed syringe coupled to liquid chromatography mass spectrometry method with hydrophilic interaction chromatography column. *Talanta* **150**, 331–339 (2016). 26838416 10.1016/j.talanta.2015.12.056

[69] Chamberlain, C. A., Rubio, V. Y. & Garrett, T. J. Impact of matrix effects and ionization efficiency in non-quantitative untargeted metabolomics. *Metabolomics* **15**, 135 (2019). 31584114 10.1007/s11306-019-1597-z

[70] Haslam, D. E. et al. Associations of network-derived metabolite clusters with prevalent type 2 diabetes among adults of Puerto Rican descent. *BMJ Open Diabetes Res. Care* **9**, e002298 (2021). 34413117 10.1136/bmjdrc-2021-002298 PMC8378385

[71] Goudo, M., Sugimoto, M., Hiwa, S. & Hiroyasu, T. The usefulness of sparse k-means in metabolomics data: an example from breast cancer data. Preprint at *bioRxiv*10.1101/2022.02.05.479235 (2022)

[72] Kim, J. O. et al. Data-driven identification of plasma metabolite clusters and metabolites of interest for potential detection of early-stage non-small cell lung cancer cases versus cancer-free controls. *Cancer Metab.* **10**, 16 (2022). 36224630 10.1186/s40170-022-00294-9 PMC9559833

[73] Mehanna, M. et al. Influence of genetic West African ancestry on metabolomics among hypertensive patients. *Metabolites* **12**, 783 (2022). 36144188 10.3390/metabo12090783 PMC9506508

[74] Li, T. et al. Longitudinal metabolomics reveals ornithine cycle dysregulation correlates with inflammation and coagulation in COVID-19 severe patients. *Front. Microbiol.* **12**, 723818 (2021). 34925252 10.3389/fmicb.2021.723818 PMC8678452

[75] Burrage, L. C. et al. Untargeted metabolomic profiling reveals multiple pathway perturbations and new clinical biomarkers in urea cycle disorders. *Genet. Med.* **21**, 1977–1986 (2019). 30670878 10.1038/s41436-019-0442-0 PMC6650380

[76] Kisuse, J. et al. Urban diets linked to gut microbiome and metabolome alterations in children: a comparative cross-sectional study in thailand. *Front. Microbiol.* **9**, 1345 (2018). 29988433 10.3389/fmicb.2018.01345 PMC6024022

[77] Rasmussen, L. G. et al. Standardization of factors that influence human urine metabolomics. *Metabolomics* **7**, 71–83 (2011).

[78] Pedley, A. M. & Benkovic, S. J. A new view into the regulation of purine metabolism: the purinosome. *Trends Biochem. Sci.* **42**, 141–154 (2017). 28029518 10.1016/j.tibs.2016.09.009 PMC5272809

[79] Khodorova, N. V. et al. Urinary medium-chained acyl-carnitines sign high caloric intake whereas short-chained acyl-carnitines sign high -protein diet within a high-fat, hypercaloric diet in a randomized crossover design dietary trial. *Nutrients* **13**, 1191 (2021). 33916877 10.3390/nu13041191 PMC8066704

[80] Nishida, Y. Relation between creatinine and uric acid excretion. *Ann. Rheum. Dis.* **51**, 101–102 (1992). 1540011 10.1136/ard.51.1.101 PMC1004629

[81] Barros, M. P. et al. Effects of acute creatine supplementation on iron homeostasis and uric acid-based antioxidant capacity of plasma after wingate test. *J. Int. Soc. Sports Nutr.* **9**, 25 (2012). 22691230 10.1186/1550-2783-9-25 PMC3439332

[82] Zyśk, B., Ostrowska, L. & Smarkusz-Zarzecka, J. Salivary adipokine and cytokine levels as potential markers for the development of obesity and metabolic disorders. *Int. J. Mol. Sci.* **22**, 11703 (2021). 34769133 10.3390/ijms222111703 PMC8584047

[83] Thirumalaikumar, V. P., Wagner, M., Balazadeh, S. & Skirycz, A. Autophagy is responsible for the accumulation of proteogenic dipeptides in response to heat stress in *Arabidopsis thaliana*. *FEBS J* **288**, 281–292 (2021). 32301545 10.1111/febs.15336

[84] Elias, R. J., Kellerby, S. S. & Decker, E. A. Antioxidant activity of proteins and peptides. *Crit. Rev. Food Sci. Nutr.* **48**, 430–441 (2008). 18464032 10.1080/10408390701425615

[85] Luan, Y.-Y. & Yao, Y.-M. The clinical significance and potential role of c-reactive protein in chronic inflammatory and neurodegenerative diseases. *Front. Immunol.* **9**, 1302 (2018). 29951057 10.3389/fimmu.2018.01302 PMC6008573

[86] Summers, K. L. & Giacobini, E. Effects of local and repeated systemic administration of (-)nicotine on extracellular levels of acetylcholine, norepinephrine, dopamine, and serotonin in rat cortex. *Neurochem. Res.* **20**, 753–759 (1995). 7566373 10.1007/BF01705545

[87] Lockman, P. R. et al. Nicotine exposure does not alter plasma to brain choline transfer. *Neurochem. Res.* **31**, 503–508 (2006). 16758358 10.1007/s11064-006-9047-5

[88] Evinger, M. J., Ernsberger, P., Regunathan, S., Joh, T. H. & Reis, D. J. A single transmitter regulates gene expression through two separate mechanisms: cholinergic regulation of phenylethanolamine N-methyltransferase mRNA via nicotinic and muscarinic pathways. *J. Neurosci.* **14**, 2106–2116 (1994). 7512633 10.1523/JNEUROSCI.14-04-02106.1994 PMC6577117

[89] Reznick, A. Z. et al. Modification of plasma proteins by cigarette smoke as measured by protein carbonyl formation. *Biochem. J.* **286**, 607–611 (1992). 1530591 10.1042/bj2860607 PMC1132941

[90] Anbarasi, K., Vani, G., Balakrishna, K. & Devi, C. S. S. Creatine kinase isoenzyme patterns upon chronic exposure to cigarette smoke: protective effect of Bacoside A. *Vascul. Pharmacol.* **42**, 57–61 (2005). 15722250 10.1016/j.vph.2005.01.003

[91] Zhu, L. et al. Spermine on endothelial extracellular vesicles mediates smoking-induced pulmonary hypertension partially through calcium-sensing receptor. *Arterioscler. Thromb. Vasc. Biol.* **39**, 482–495 (2019). 30626206 10.1161/ATVBAHA.118.312280

[92] Wang, C. et al. Nicotine accelerates atherosclerosis in apolipoprotein e-deficient mice by activating α7 nicotinic acetylcholine receptor on mast cells. *Arterioscler. Thromb. Vasc. Biol.* **37**, 53–65 (2017). 27834689 10.1161/ATVBAHA.116.307264

[93] Pesci, A. et al. Mast cells in the airway lumen and bronchial mucosa of patients with chronic bronchitis. *Am. J. Respir. Crit. Care Med.* **149**, 1311–1316 (1994). 8173772 10.1164/ajrccm.149.5.8173772

[94] Feng, P. et al. Human supplementation with *Pediococcus acidilactici* GR-1 decreases heavy metals levels through modifying the gut microbiota and metabolome. *NPJ Biofilms Microbiomes* **8**, 63 (2022). 35974020 10.1038/s41522-022-00326-8 PMC9381558

[95] Booth, S. C., Workentine, M. L., Weljie, A. M. & Turner, R. J. Metabolomics and its application to studying metal toxicity. *Metallomics* **3**, 1142–1152 (2011). 21922109 10.1039/c1mt00070e

[96] Deng, P. et al. Application of metabolomics to characterize environmental pollutant toxicity and disease risks. *Rev. Environ. Health* **34**, 251–259 (2019). 31408434 10.1515/reveh-2019-0030 PMC6915040

[97] Fu, Z. & Xi, S. The effects of heavy metals on human metabolism. *Toxicol. Mech. Methods* **30**, 167–176 (2020). 31818169 10.1080/15376516.2019.1701594

