# Supplementary material for: The salivary metabolome of children and parental caregivers in a large-scale family environment study
Source: NPJ Metab Health Dis. 2024 Aug 13;2:22. doi: 10.1038/s44324-024-00024-3 (PMC12118755; doi:10.1038/s44324-024-00024-3)
Supplement: Supplementary file 2 — Supplemental_information [file 44324_2024_24_MOESM2_ESM.pdf]

Supplemental material for: The salivary metabolome of children and parental caregivers in a large-scale family environment study

Jason A. Rothman[1,2,3#], Hillary L. Piccerillo[2], Sage J.B. Dunham[1], Jenna L. Riis[2,4], Douglas A. Granger [2,5,6], Elizabeth A. Thomas[2,7], and Katrine L. Whiteson[1,2#]

[1]Department of Molecular Biology and Biochemistry, University of California, Irvine, Irvine, CA, 92697

[2]Institute for Interdisciplinary Salivary Bioscience Research, University of California, Irvine, Irvine, CA, USA

[3]Department of Microbiology and Plant Pathology, University of California, Riverside, Riverside, CA, USA

[4]Department of Health and Kinesiology, University of Illinois Urbana-Champaign, Urbana, IL, USA

[5]Department of Psychological Science, University of California, Irvine, Irvine, CA, USA

[6]Department of Pediatrics, Johns Hopkins University School of Medicine, Baltimore, MD, USA

[7]Department of Neurobiology and Behavior, University of California, Irvine, Irvine, CA, USA

[#] **Co-corresponding authors**: Jason Rothman, Department of Molecular Biology and Biochemistry and Institute for Interdisciplinary Salivary Bioscience Research, University of California, Irvine, Irvine, CA, 92697, rothmanj@uci.edu, (949) 824-3509; Katrine Whiteson, Department of Molecular Biology and Biochemistry and Institute for Interdisciplinary Salivary

Bioscience Research, University of California, Irvine, Irvine, CA, 92697, katrine@uci.edu, (949) 824-9032.

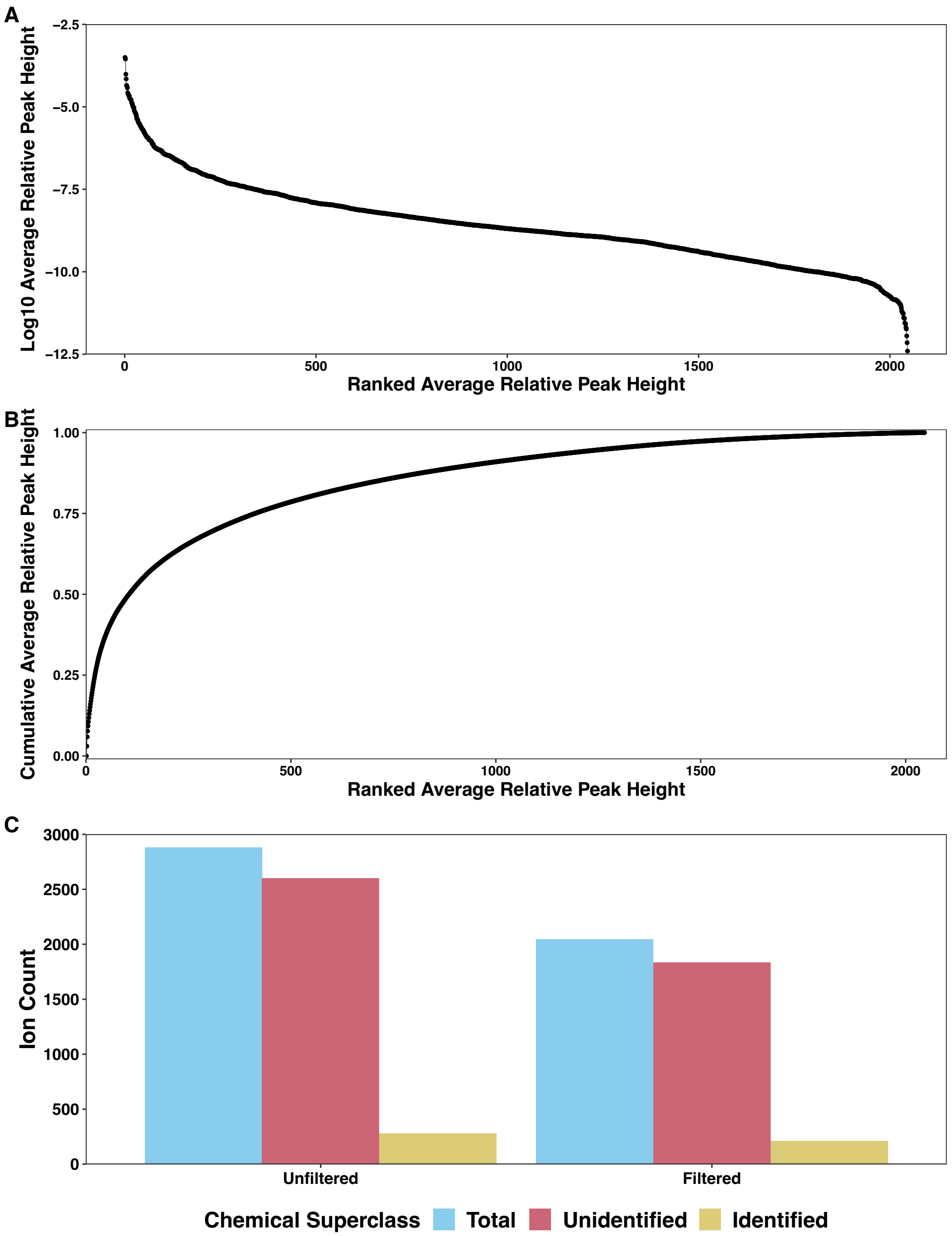

Figure S1: A) Rank abundance curve of the $\log_{10}$ transformed average relative peak heights of ions across all samples. B) Accumulation curve of the ranked average relative peak heights of ion across all samples. C) Counts of unique ions before and after filtering out ions that were less than two-fold higher in relative peak height as compared to blank samples. Color denotes total ion count, and if the ions were named or unidentified.

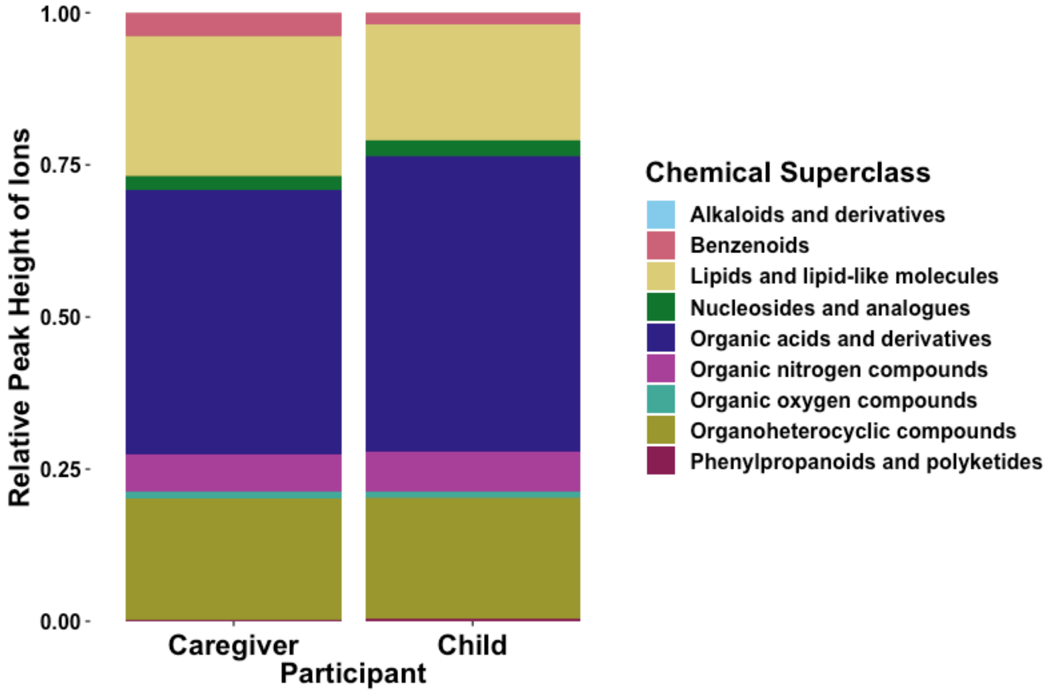

Figure S2: Stacked bar plot showing the average relative peak heights of the chemical superclasses of 281 identified ions grouped by participant as assigned by ClassyFire. Color denotes the chemical superclass.

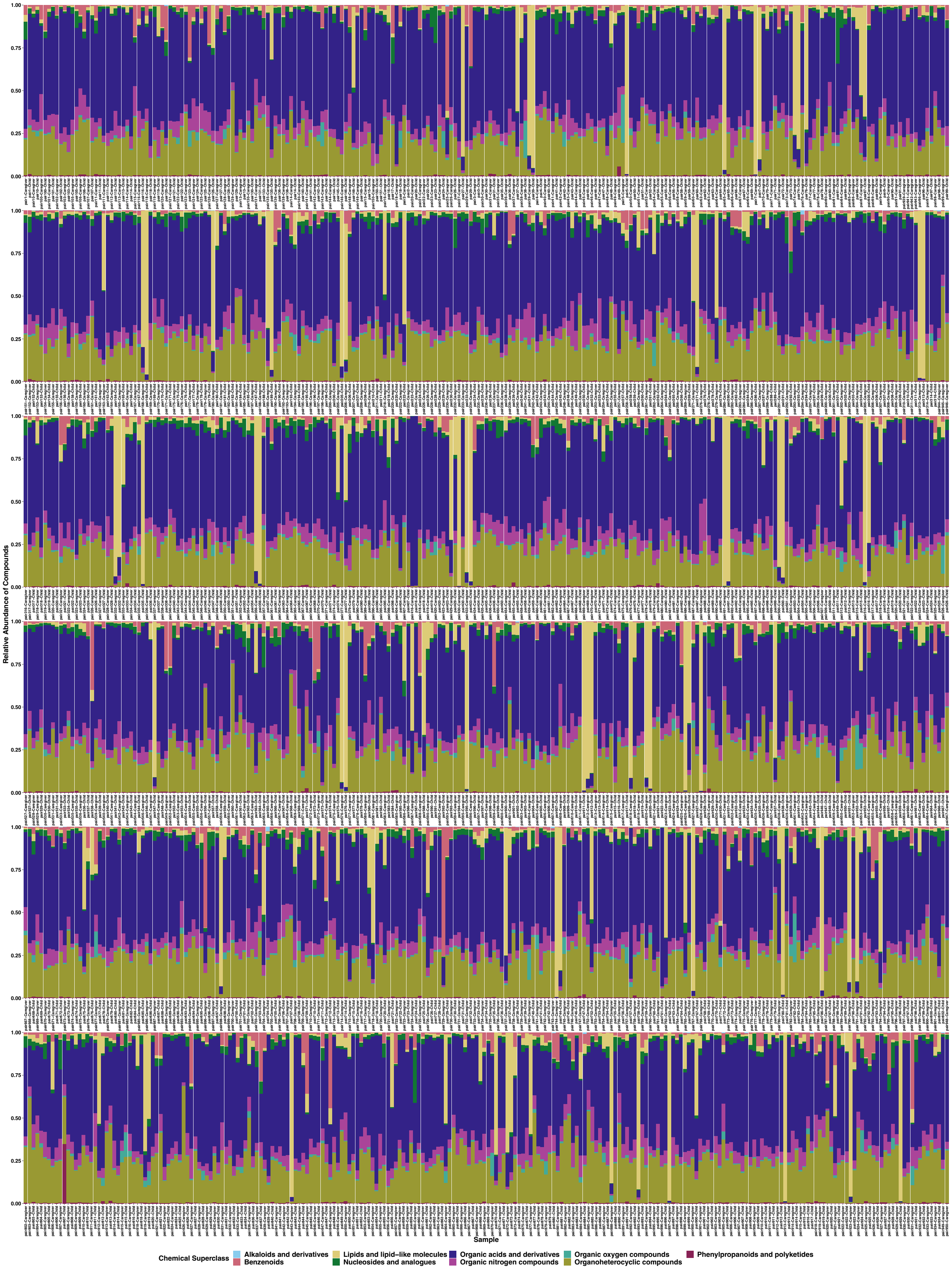

Relative Abundance of Compounds
1.00
0.75
0.50
0.25
0.00
Sample
Chemical Superclass
Alkaloids and derivatives
Benzenoids
Lipids and lipid−like molecules
Nucleosides and analogues
Organic acids and derivatives
Organic nitrogen compounds
Organic oxygen compounds
Organoheterocyclic compounds
Phenylpropanoids and polyketides

Figure S3: Stacked bar plot showing the relative peak heights of the chemical superclasses of 281 identified ions across samples as assigned by ClassyFire. Color denotes the chemical superclass.

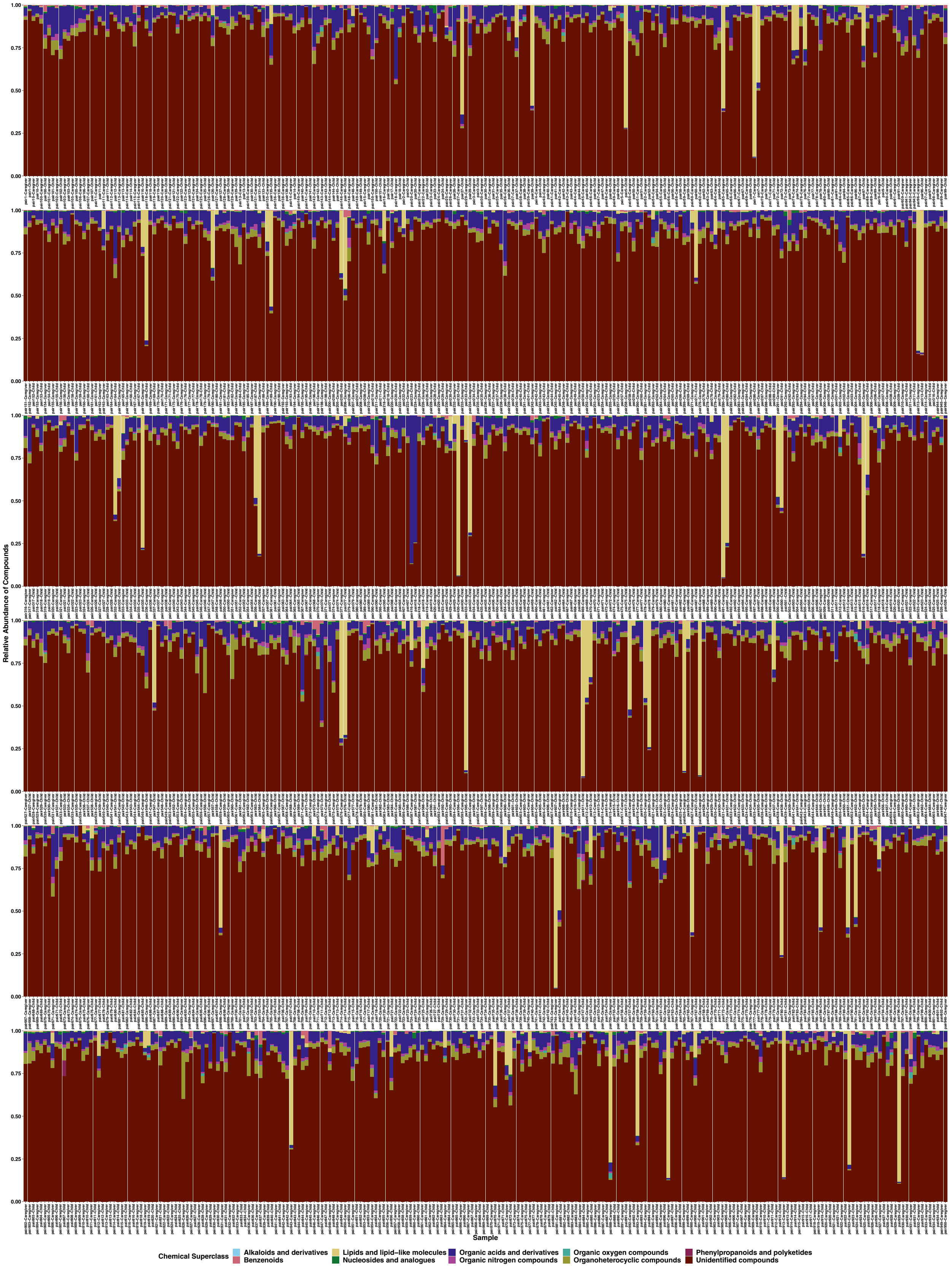
Relative Abundance of Compounds
Sample
Chemical Superclass
Alkaloids and derivatives
Benzenoids
Lipids and lipid−like molecules
Nucleosides and analogues
Organic acids and derivatives
Organic nitrogen compounds
Organic oxygen compounds
Organoheterocyclic compounds
Phenylpropanoids and polyketides
Unidentified compounds

Figure S4: Stacked bar plot showing the relative peak heights of the chemical superclasses of all ions across samples as assigned by ClassyFire. Color denotes the chemical superclass and unidentified ions.

Supplemental dataset SF1: Relevant statistical information for all Spearman correlation tests and linear models. Each worksheet tab contains information for individual tests. “Padj” denotes p-values adjusted for multiple comparisons.
